# Collective excitations of germinating pollen grains at critical points

**DOI:** 10.1038/s41598-023-27754-6

**Published:** 2023-01-12

**Authors:** Mariusz A. Pietruszka

**Affiliations:** Faculty of Natural Sciences, Institute of Biology, Biotechnology and Environmental Protection, The University of Silesia, 28 Jagiellońska St., 40032 Katowice, Poland

**Keywords:** Biological techniques, Biophysics, Cell biology, Computational biology and bioinformatics, Physiology, Plant sciences, Systems biology, Climate sciences, Environmental sciences, Planetary science, Physics

## Abstract

In plants, the germinating pollen grain (pollen tube) is a single, elongated cell that serves as a conduit through which gametes pass. Pollen tubes display a fast growth rate, which under certain conditions, changes periodically and is accompanied by ion exchange with the growth environment. Therefore, pollen tubes exposed to various abiotic conditions may adversely affect or improve their reproductive performance and fertility. We examined a collection of live pollen tubes of tobacco (*Nicotiana tabacum* L.) and hyacinth (*Hyacinthus orientalis* L.) using a non-invasive semiconductor–electrolyte interface technique in the vicinity of the germination temperature or optimum growth temperature of a pollen grains/tubes. The time series measurements and numerical calculations, performed using information theory methods, represent signatures of collective dynamics in living cells at critical—molecularly encoded—germination and growth temperatures. This method (and soil pH data) can facilitate assisted plant migrations from one ecosystem to another as the Earth faces climate change.

## Introduction

This article examines the essential physical variables that can be a manifestation of complex biological phenomena. Dynamic entropy^1^ and other dynamic metrics that may comprise the complexity of living systems are the variables that can provide new insight into fundamental biological processes or functions at the molecular, inter-cellular, or organism level. Henceforth “dynamic” means derived from the time series, a series of real numbers, while extracellular pollen tube ions form an interacting nonlinear system.

Based on quantitative experimental data, statistical physics methods, and numerical calculations, a temperature-driven re-entrant non-equilibrium phase transition, manifested in a Lorentz resonance, occurred in the fluctuation amplitude of the extracellular ion fluxes of a collection of metabolically active pollen tubes at a critical point. This finding may interest individuals in cell biology and plant physiology, as well as in non-equilibrium statistical physics and biological physics, not to mention its potential use in assisted plant migration as the Earth struggles with climate change.

The research method, based on a previously published semiconductor–electrolyte technique^2–5^, enables the precise determination of individually variable molecularly coded critical (meaning germination or optimal plant growth) temperatures that occur in the form of Lorentz resonances in biological systems. We detected the critical temperatures by observing the change in the ionic noise near the point of the non-equilibrium phase transition. The presented method can be used to study the influence of various external factors, including treatment with phytohormones or ion channel inhibitors, on cell growth. Combined with single-cell transcriptome sequencing, this technique provides scientists with new tools to study cell growth at the molecular and biophysical levels.

Quasi-particles (or collective excitations) are emergent phenomena that arise when a microscopically complex system behaves collectively at a critical temperature. From the point of view of an outside observer, they are usually considered as a macroscopic coherent state. Although quasi-particles occur in several many-body systems in physics, little is known about their occurrence in biological systems. Here we show that quasi-particles can also exist in a non-equilibrium phase transition at a critical temperature, where they can act as a long-term information carrier inside the system of cells.

Germinating pollen grains are cells growing at their end that is highly polarized, i.e., elongated in one dimension to allow the transport of gametes during fertilization. Polarized growth occurs via the apical expansion of the pollen tube, which requires the deposition of new cell wall material at the apex. Pollen tube growth depends on a complex mechanism that integrates various molecular and cytological sub-processes. Recently, these cells’ signaling and development have been reported to depend on the cytosolic pH gradients^6^. In contrast, the plasma membrane proton (H^+^) ATPases (AHAs) have been proposed to promote pollen tube growth and participate in cell polarization. These findings define AHAs as energy transducers that support the ionic circuit, representing the spatial and temporal cytosolic pH profiles, thereby controlling the pH-dependent mechanisms necessary for pollen tube elongation and, thus, for plant fertility. Recent theoretical works^7,8^ proposed a pH- and conjugated temperature-dependent growth mechanism for plant cells and the primary wall of grasses. Therefore, a similar mechanism can be anticipated in pollen tubes for generic reasons. However, the problem of the mechanism that leads to the intensification (amplification, enhancement) of the correlation of the H^+^ (hydronium) ions—and thus lower the pH level—near to and at the characteristic (critical) growth temperature for a given plant species remains unidentified. It seems that apart from the apparent biological aspect, it is also a problem known in condensed matter physics as a many-body problem and, therefore, should be solved using the advanced methods of statistical physics.

Chemical potential is energy that can be absorbed or released due to a change in the number of particles; the chemical potential is the Gibbs free energy per particle. As with other thermodynamic potentials, rapid changes in the chemical potential locate the critical temperatures in the studied condensed matter system in phase transitions^9–12^. These phase transitions can be detected by proximity effect using the contact electrode method^13^. We proposed a similar contact method to determine the level of chemical potential in the soft matter for the ion oscillations of a single (living) cell using the n-type semiconductor–electrolyte interface^2^. The latter also enabled the localization of the characteristic temperature of individually variable human peripheral blood and the detection of non-equilibrium phase transition^4,5^, respectively. In this article, we verify and confirm the existence of a noise-induced (or -detected) non-equilibrium phase transition^14^ experimentally in a, generating ionic fluxes, ensemble of pollen tubes. By detecting the critical growth temperature, the pink/white noise revealed an ordered symmetry-breaking state through a genuine phase transition at criticality.

In non-equilibrium systems (here: growing pollen tubes), the critical point is the attractor of the dynamics that lead to self-organized criticality^3,15^. This orchestrated instability can be achieved through long-range correlations that ultimately lead to phase coherence in the system. Here, we tested the hypothesis of whether elongating pollen tubes undergo a specific cooperative behavior—collective excitations^16^—and generate the increased ionic fluxes observed as the macroscopic voltage fluctuations at the critical temperatures. The latter is vital for all of the metabolic processes as well as for overall cell homeostasis.

Based on experiments and numerical calculations, the critical temperature was precisely determined. A range of temperatures close to the germination or optimum growth enabled this method to determine these cardinal features in various species. Together with the available literature on soil pH, these precisely calculated data can be extrapolated to sort out plant migration from one ecosystem to another.

We apply Occam’s razor principle to avoid endless expansion of the volume of this report:

1. Mainly, the biophysical/biochemical aspect of the phenomenon is considered.
2. The work starts from the assumption that the central system of the pollen tube is proton flux and the role played by the proteins that determine it, whereas this is only partially true. The proton flux is only one of the components used to guide the pollen tube. For example, the role played by calcium or chloride ions cannot be ignored; one must include the role played by other cellular components. Therefore, to save space, only a new phenomenon has been discussed and its potential use in plant science and beyond.

## Results

### Distinct identities of pollen tubes revealed by measurements of the electromotive force

**Figure 1:**
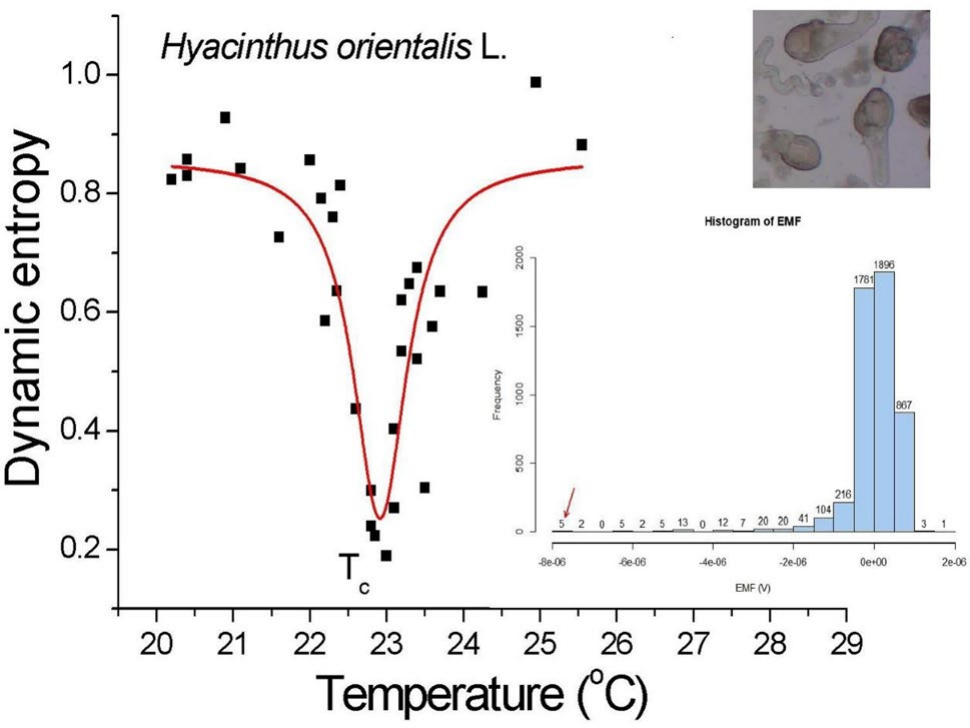
Dynamic entropy of hyacinth pollen tubes as a function of temperature and **Inset** fat-tailed electromotive force (EMF) histogram at the critical temperature $T_{c}$. Entropy (ApEn) was *calculated* (each point separately) for the empirical EMF time series that was induced by the extracellular ionic fluxes of an isolated (intact) droplet that contained a collection of hyacinth pollen tubes. The determination coefficient for the experimental data that was re-calculated in R language (series of 5000-time points for each temperature at 30 different temperatures; sampling rate 4.1 Hz) fitted to the Lorentz resonance curve equaled R^2^ = 0.7583 (χ^2^ = 0.014); half-width w_1/2_ = 0.83(18) °C. The uncertainty of the individual temperature measurements equaled $\Delta T = 0.1$ °C. Note the extreme value of 5 × (− 8) × 10^−6^ V ≈ 5 × (− 1) × 10^−5^ V (i.e., more than two orders of magnitude greater than the noise level) in the inset (red arrow)—Photomicrograph of the elongating pollen tubes of *Hyacinthus orientalis* L.

**Figure 2:**
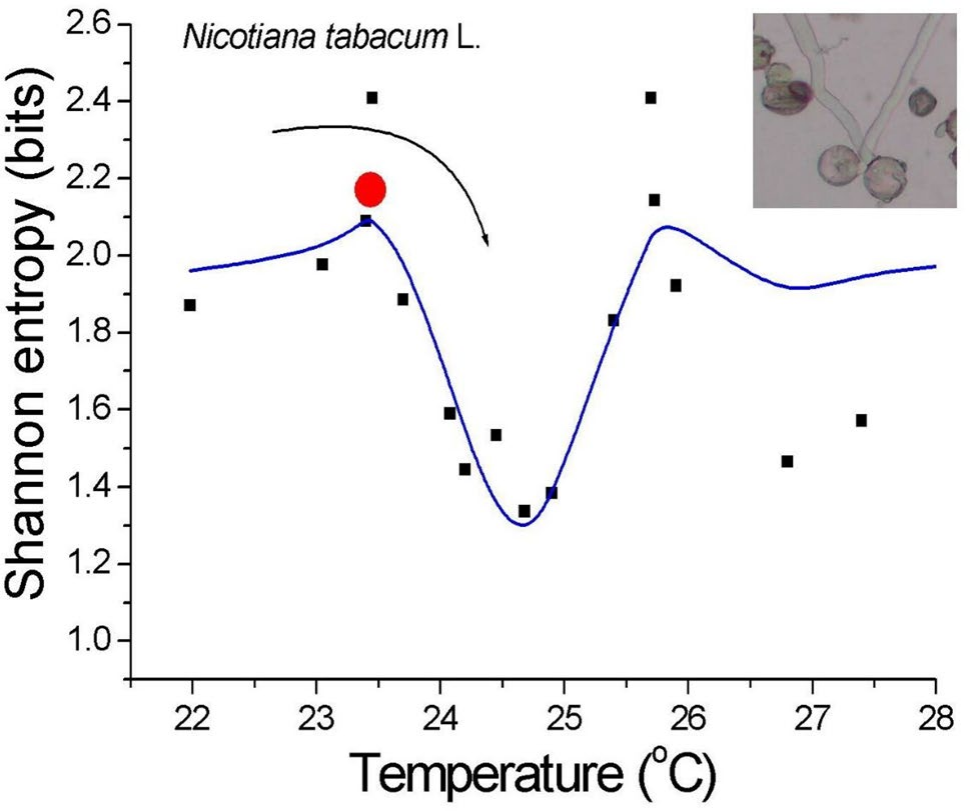
Dynamic Shannon entropy as a function of temperature for living tobacco pollen tubes. The data points (solid squares), calculated separately in the R programming language from the original time series, were interpolated using the sinc function and showed a zero-bias peak and splitting (bound state) at criticality. The red ball with the arrow, representing thermally activated proton over-the-barrier hopping dynamics, points to the attractor basin, which can be interpreted as a 2H^+^ ion(s) energy gap ($\Delta$) that was approximately 2.25 K (0.00019(1) eV) wide, which indicated the readiness of the pollen tubes for an extreme (fast) and stable elongation at a critical range. Note the clear local maxima that can be interpreted as potential barriers (the system can tunnel through them to achieve a stable minimum), which also represent points of instability. Fit parameters: coefficient of determination R^2^ = 0.62587 (χ^2^ = 0.057), y_0_ = 1.95(8), a = − 0.19(5), b = 3.57(6); $y = y_{0} + \frac{{a {\text{sin}}\left( {bx} \right)}}{x}$. Photomicrograph of the elongating pollen tubes of *Nicotiana tabacum* L.

**Figure 3:**
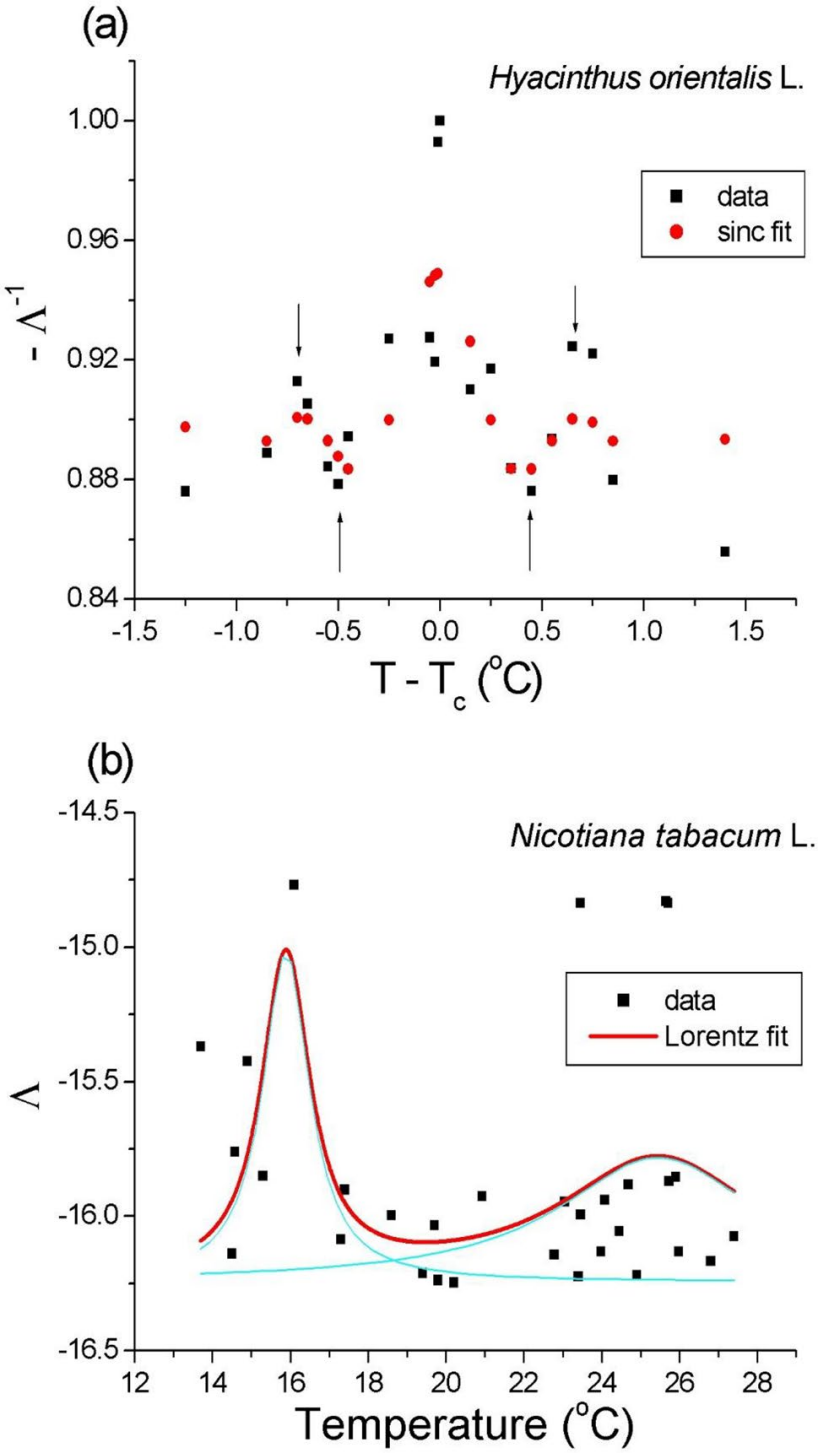
Dynamic range of the calculated dominant Lyapunov exponent (Λ, log scale) as a function of temperature for the hyacinth (**a**) and tobacco (**b**) pollen tubes at criticality. Fit parameters to the sinc function (red dots): R^2^ = 0.51 (χ^2^ = 0.0005), baseline y_0_ = 0.894(5), $a$ = 0.05(1), and $b = 11.27 \left( {143} \right)$; $T_{c} \cong 22.85$ °C. (b) Two critical regions were indicated by the maxima of the multi-peak Lorentz resonance curve: germination at 15.89(45) °C and optimum at 25.44(193) °C. Data points (solid squares) from the experimental time series (each point represents 5000 measurements) were calculated in R code.

**Figure 4:**
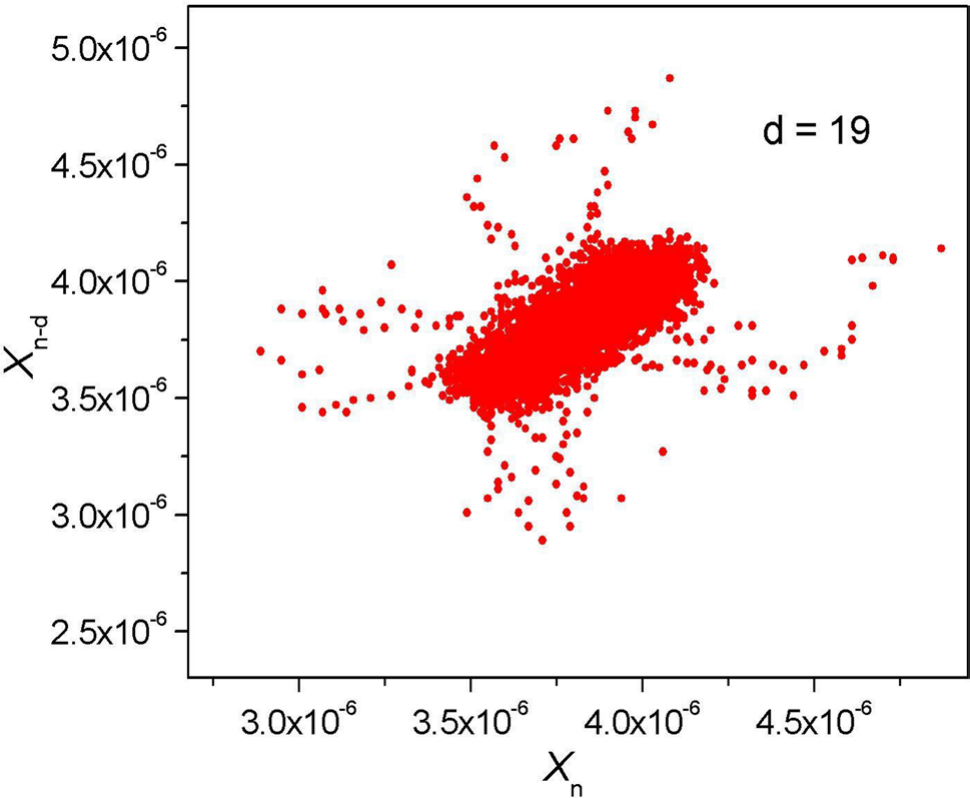
Butterfly attractor (calculated Poincaré section) for the germinating pollen grains of hyacinth on the transition from germination to the optimal growth temperature showing the cyclicality ($d = 19$) in this chaotic system, which indicate that a short-term ~ 19/(4.1 Hz) = 4.64 ± 0.5 s oscillation is the fundamental frequency ($f_{0} \approx 0.23$ Hz) in the system.

**Figure 5:**
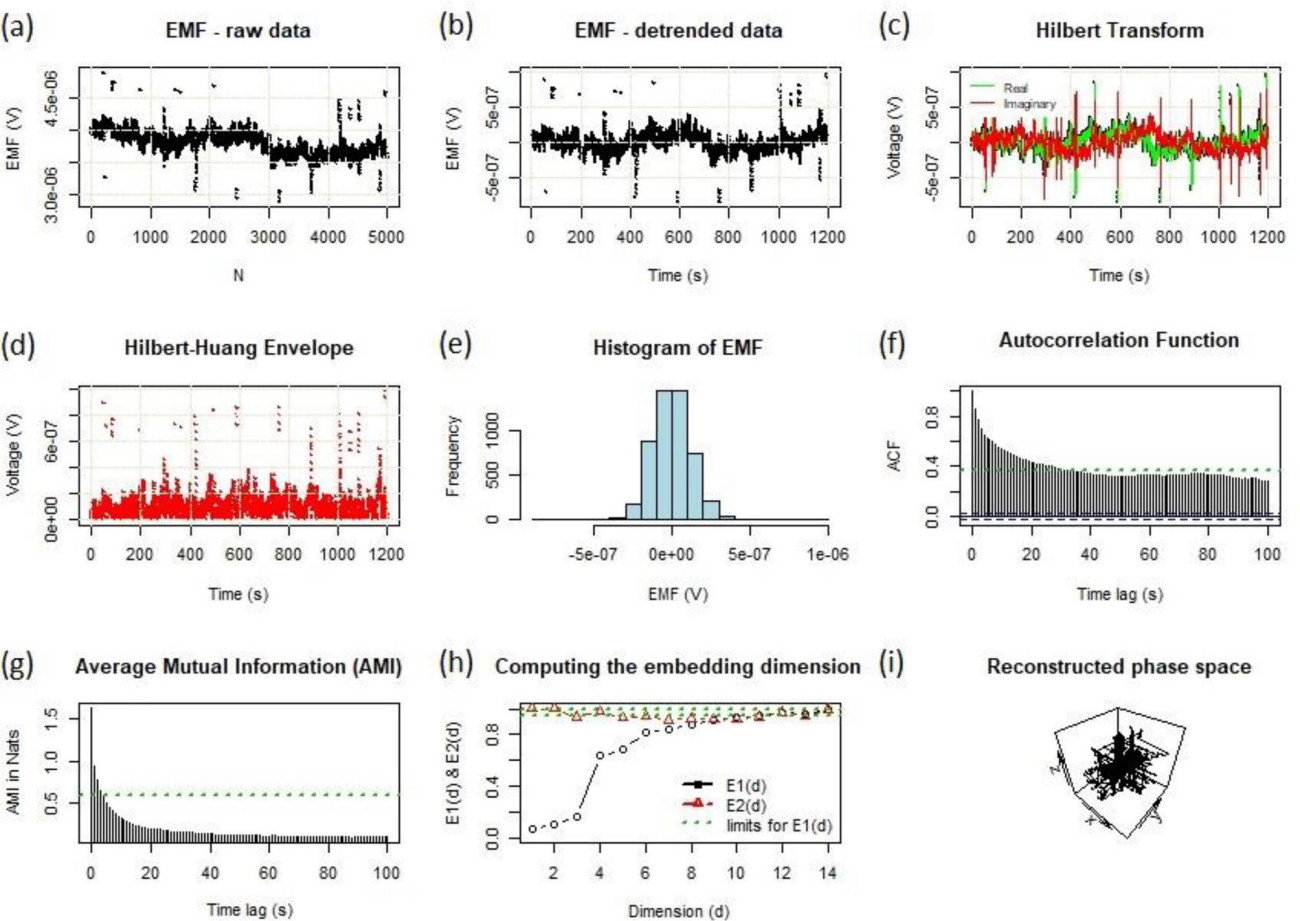
Different complexity measures of a collection of hyacinth pollen tubes at temperatures *different* from the optimum growth temperature ($T \ne T_{c}$) close to 20.2 °C. (**a**) Electromotive force (EMF) as a function of the point counter ($N$). [EMF can be measured as an open circuit potential difference or voltage that can drive an electric current if an external circuit is connected to the terminals]. (**b**) Detrended data from (**a**). (**c**) Hilbert transform. (**d**) Hilbert–Huang envelope. (**e**) Histogram of EMF. (**f**) Autocorrelation function and (**g**) Average mutual information as a function of the time delay. (**h**) Computing the embedding dimensions. (**i**) Reconstructed phase space trajectory according to Takens' theorem. Representative chart from 62 measurement series.

**Figure 6:**
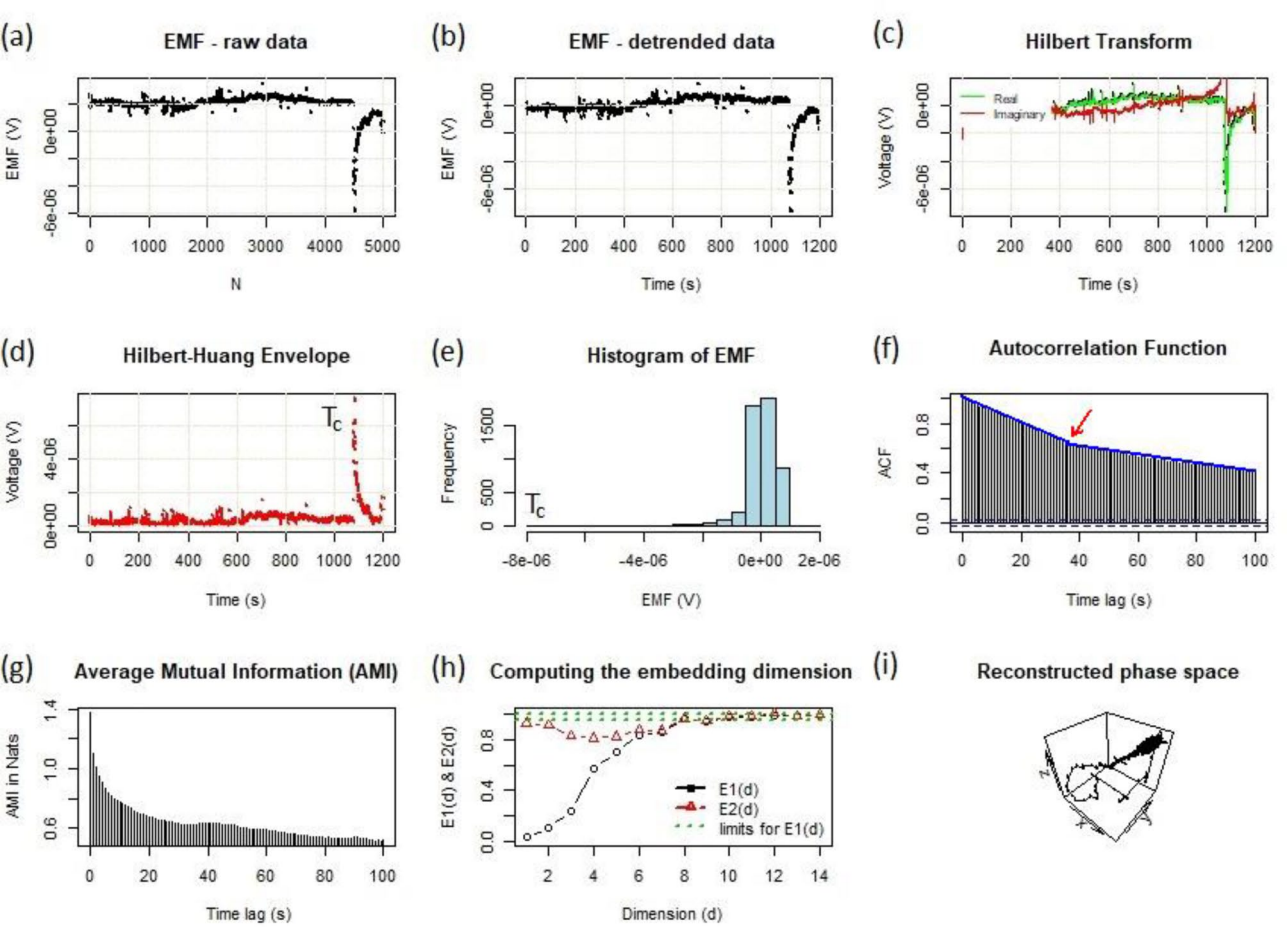
Different complexity measures of the collection of hyacinth pollen tubes at temperatures close to the optimum growth temperature ($T \cong T_c$). The thermal excitation in this dissipative system occurred at the critical point (indicated) when the resonance conditions were met. Note the bilinear character (blue lines of different slope) in panel (**f**), which show a characteristic time delay (red arrow) of approx. 38–40 s, a value that is characteristic of the oscillation of a pollen tube.

**Figure 7:**
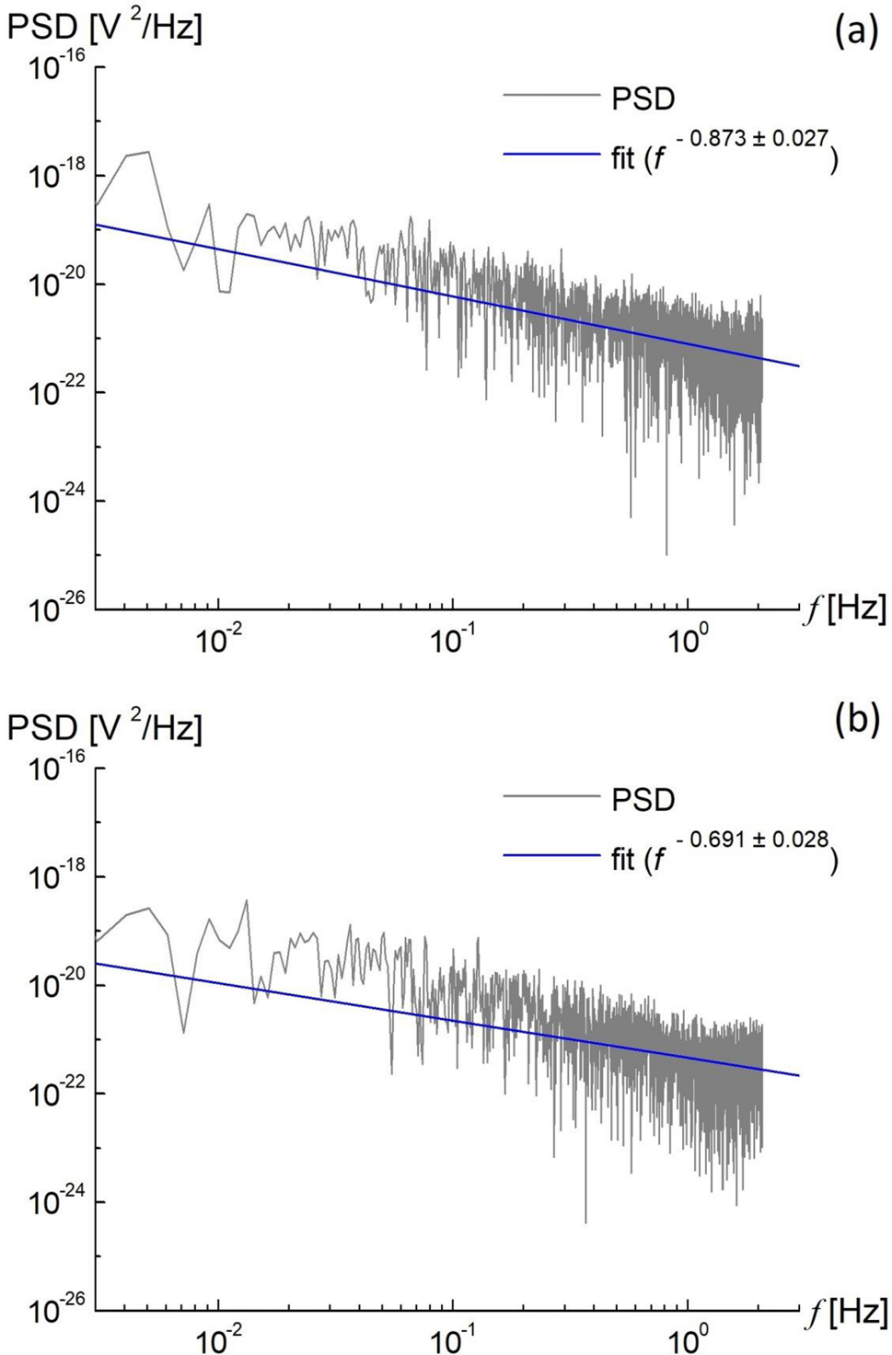
Power spectral density (PSD) as a function of the frequency ($f$) of the extracellular ionic fluxes of hyacinth at (**a**) 22.95(5) °C and (**b**) 20.20(5) °C. The calculated linear descent of the spectral exponent ($\beta$) equaled (**a**) 0.873(27) and (**b**) 0.691(28) and represented (**a**) pink and (**b**) pink/white noise, respectively. Note that the linear, logarithmic curve (**a**) signals the relationship with the coherent dynamics, whereas the spectral exponent corresponds to the fractal dimension of the system dynamic.

We investigated a collection of live pollen tubes of tobacco (*Nicotiana tabacum* L.) and hyacinth (*Hyacinthus orientalis* L.) using a non-invasive semiconductor–electrolyte interface technique (ELoPvC^2,3^) at different temperatures located near to and at the critical growth temperatures. We present the results of our research in Figs. 1, 2, 3, 4, 5, 6 and 7 and Table 1. The data flow diagram for a single measurement in auxiliary Fig. S1 and R code listing in Fig. S2 supplements the latter.

**Table 1:** Complexity metrics of a droplet of the electrolytic medium containing a collection of the living pollen tubes of hyacinth at a critical (optimum) growth temperature and beyond.

Meas. #* (°C) hyacinth	Corrected R/S Hurst exponent	Empirical Hurst exponent	Entropy ChaoShen (in bits)	Entropy ApEn SampEn	Lyapunov exponent (Λ) [emb. dims]	Spectral exponent (β)
00249 @22.95(5) critical	0.96	0.96	1.41 (2.04)	0.1427 0.1037 Deterministic	− 15.26 [10]	0.873 (27)
00232 @20.20(5) beyond	0.87	0.89	1.69 (2.44)	0.8240 0.7528 Random	− 16.14 [10]	0.691 (28)

*Raw data files are available upon reasonable request.

In Figs. 1, 2 and 3, Lorentz resonances localize two critical temperatures: germination and optimum (maximum) growth. Figure 1 shows the calculated dynamic entropy^1^ of the hyacinth (see “Materials and methods” Section) from the experimental data series obtained in our experiment as a function of temperature. In Fig. 1, a deep minimum of entropy occurred at approximately 23 °C. The latter value agrees with the literature data for the optimum (or critical) growth. The resonance peak corresponded to the critical fluctuations^17^ in this non-equilibrium system. The critical temperature (T_c_) for minimum entropy at 22.95(5) °C comprises three phases: subcritical (below T_c_, time-dependent, irreversible), critical (at T_c_, timeless, quasi-reversible), and supercritical (above T_c_, time-dependent, irreversible), indicated a non-equilibrium phase transition, by increasing the size of the correlated coherent domain, in a system of metabolically active cells. On the other hand, a sharp entropy minimum identified the *molecularly coded* value of the optimum (or critical) growth temperature. Then we used the histogram in the insert to Fig. 1 to calculate Shannon's information entropy^18^ to show that *different* complexity metrics can recognize the extremum value at T_c_.

The dynamic Shannon entropy is the average amount of information that can be conveyed by an event when considering all of the possible outcomes. It is plotted as a function of temperature for living tobacco pollen tubes, as shown in Fig. 2. The calculated data points were interpolated using the sinc function, which shows the zero bias peak and splitting at criticality. [Note that the sinc function is the Fourier transform of the rectangular function, which in the limit is the Dirac delta function]. The lowest value that was obtained for T_c_ in a binary representation (b = 2) was close to unity, which can be interpreted as the whole system can be described as being in only one state of “zero” (or “one”). Beyond criticality, the system became unstable (see the red ball at the edges of the well for the mechanical analog in Fig. 2). It was vulnerable to even small internal or external perturbations. Conversely, the bound state that arose in the center of the gap becomes immune to energy disturbances less than 2 × 10^−4^ eV (< 2 K). This important finding is in line with our daily observations—slight changes in the ambient temperature around the optimal growth temperature do not cause significant changes in plant growth.

The dynamic range of the dominant Lyapunov exponent (Λ) as a function of temperature is shown in Fig. 3. Note the change of Λ of approximately two orders of magnitude in the dynamic range for the hyacinth at critical temperatures and compare it with the theoretical predictions presented in Ref.^19^. A similar, however, less pronounced result was obtained for the tobacco pollen tubes. The dynamic range was approximately one order of magnitude for germination or the optimal growth temperatures, which means that the system underwent a phase transition at both critical temperatures. However, the raw data seemingly showed the enhancement of fluctuations (critical fluctuations) around the two crucial points (compare with Fig.  5 in Ref.^14^ where σ^2^ is related to temperature in this paper) as in Fig. 3.

The Poincaré Sect.^20^ of the form ($$x_{n} , x_{n - d} )$$, where the value of *d* is selected experimentally at the peak system oscillations, was also considered. The resulting attractor, presented in Fig. 4, is butterfly-shaped, although it was weakly expressed against the background of chaotic behavior. The individual pulses of the electromotive force were most likely responsible for its appearance. As a result, cyclicality was present in the system but was weak. The butterfly effect describes the mixing of information in a (quantum) chaotic system. In the presented case, the calculations revealed an interesting result—the oscillation provides the fundamental (excitation) frequency ($$f_{0}$$) in the system, which can roughly be compared to the H^+^ flux oscillations that were obtained in Ref.^21^. This is consistent with the proposal^6^ that the membrane proton (H^+^) ATPases promote pollen tube growth.

This picture is reinforced by the nonlinear complexity metrics of the ensemble of hyacinth pollen tubes at temperatures that differ from the optimum growth temperature (T ≠ T_c_), as shown in Fig. 5. From (a) electromotive force data through (b) detrended data from (a), (c) Hilbert transform, and (d) Hilbert-Huang envelope, we end up with the (e) histogram of the EMF. It turned out that the histogram was a symmetric Gaussian beyond T_c_. Moreover, the autocorrelation function, Fig. 5f, shows a distribution with a fat tail, which means that the system was not correlated or was weakly correlated. The average reciprocal information distribution (g) also confirmed the latter. The calculation of the embedment dimension (h) enabled us to reconstruct the phase space of our system according to Takens’ theorem. It turned out that the calculated from (b) phase space trajectory was spherically symmetric, i.e., isotropic, which means that the investigated system can be found at any point in this parameter space with an (almost) equal probability.

The situation was different in criticality (T = T_c_), where there was a synchronous action of germinating pollen grains, as is shown in Fig. 6. Here, the other nonlinear complexity metrics revealed a distinct extreme that identified the critical point. All of the panels (a)–(d) had a specific peak (a short-time instability), whereas the histogram (e) was highly asymmetric. Moreover, the autocorrelation function (f) was a (bi)linear function. We observe a linear suppression of the autocorrelation function, which means that near the critical point, there were long-range correlations (compare with Fig.  7 in Ref.^17^ for a critical behavior), which is a characteristic feature of a phase transition. The transition from dissipative to a phase with coherent (superfluid) behavior (frictionless ion flux, non-dissipative, which should exceed the one that arises from diffusion or active transport^22^ with no affinities, no gradients of temperature and no gradients of chemical potential), apparently occurred at criticality (T_c_). This situation was reflected in the reconstructed phase space (i), which shrank to a point. Moreover, the *molecular coding of the optimal temperature of life as a benchmark for homeostasis* was identified through the direct observation of the spontaneous electric voltage (Fig. 6a), which peaked at T_c_. It provides evidence for the existence of cooperative phenomena (collective excitations) that multicellular systems should benefit from.

The scale-free behavior independently confirmed these results in power spectral density (PSD), calculated as a function of the frequency of the extracellular ion fluxes in our system, Fig. 7. Similar to Ref.^3^, the pink noise spectral exponent, also known as 1/f or flicker noise denoting signal or process in a frequency (f) spectrum whose power spectral density is inversely proportional to frequency, was close to 1 at criticality. In contrast, the linear descent of the spectral exponent β was closer to 0.5 and had a weakly correlated pink/white (or Gaussian) noise beyond T_c_. A usual isomorphism between fractal self-similarity and coherent states is established. Note that the fractal dimension is given by the angular coefficient of the straight line in the log–log plots. The linear functional logarithmic dependence of the spectral power density on frequency, which typically characterizes a fractal self-similarity, signaled that a coherent state dynamic was at the heart of the system at the critical point (Fig. 7a). In other words, we are approaching sensing the waves in intercellular communication through their effect on electromotive force. Such a feature (pink noise) we expect when infrared signals -with a long wavelength or low frequencies—are beginning to emerge (see also Fig. 2 in Ref.^3^).

The complexity measures of the electrolytic medium droplets, which contained a small community (~ 50) of living hyacinth pollen tubes at the critical growth temperature and beyond, are also listed in Table 1. The corrected R/S (empirical) Hurst exponent used to measure a time series’s long-term memory was closer to unity at the critical conditions as was expected. The system was also more deterministic at T_c_ than outside of this area.

## Discussion

New theoretical models or experiments usually raise many questions. However, a general question is how we can interpret the non-equilibrium temperature in the context of the proposed experiments. We know that classical thermodynamics or statistical mechanics defines the physical temperature in the results. It is the same as the one on which state functions are based and from which the phase diagrams of substances are derived, which are strictly subject to the thermodynamic equilibrium. However, when the system is unbalanced, there is no single temperature^5^, i.e., there can be many temperatures in the non-equilibrium system. Thus, we can speak of a temperature in a non-equilibrium system only when such a system is locally in thermal equilibrium. However, the temperature can be determined at any time (isochoric cut) as long as the system is locally at equilibrium. In our experiment, we can interpret the displacement temperature as the slowly changing mean ambient temperature in contact with the sample at any time in the time series. Thermal fluctuations^23^ give rise to an extremely specific (critical) temperature, i.e. when resonance conditions are met, at which there is a phase change in the extracellular ion streams of the pollen system, see Fig. 6d–f. The transition can only be noticed if not too large temperature ranges are assumed between consecutive measurements. Minute temperature variations help to establish the correct transition temperature to some extent, as is indicated in Fig. 6d, where the equality of the local (sample at the point of transition) and ambient temperature was achieved.

Moreover, the ‘breakdown’ that appears in Fig. 6f after approx. 38–40 s (time delay) can be interpreted as a secondary transition in the system that presumably reflects the transport of the Ca^2+^ and Cl^−^ ions or the H^+^/K^+^ co-transport (review, e.g., Ref.^24^ and papers cited therein). However, this exciting result requires further research.

In this report, while all of the complexity measures appear to be built on different assumptions, the results presented tell us the same story—the peaks in Figs. 1, 2 and 3 clearly show the enhancement of the fluctuations around the critical points. Does this result from any particular feature of the critical point, or do they generally converge to obtain the same effect? The answer seems to be both—they coincide with the same result. Moreover, the calculated PSD function showed that the spectral exponent at criticality was close to unity (Fig. 7a). For normalization purposes, every time series were 5000 points long to yield the calculated measures. At first glance, the appropriate period (here: 20 min) can be shortened, and the sampling rate can be increased. However, an isochoric truncation, which is the boundary of this process, leaves us with only one point, and it becomes impossible to calculate the dynamic entropy and other traits; this uncharted problem alone can also become an intriguing research task.

Ultimately, intense competition^25^ between a community of growing pollen tubes may lead to cooperative behavior (and consequently increased fertility) under favorable temperature conditions. Already in Ref.^26^, the results indicated an accelerating effect of temperature increase on pollen germination and pollen tube growth kinetics, as well as an increase in the number of pollen tubes that reach the style base. The commonly accepted view of periodically oscillating pollen tubes seems inadequate for the collection of pollens. However, it can be fulfilled under critical conditions (Table 1) when a system goes from chaotic to deterministic. Moreover, as seen through a magnifying glass, the macroscopic (wave) behavior of a cell system reflects the rhythm of the ion fluxes for a single pollen tube and the fact that the system reaches the favorable evolutionary state with the lowest energy dissipation at the critical point.

### Assisted plant migration: potential application

Global warming is causing a progressive increase in ambient temperature. Plants, sedentary organisms, are at risk from these changes. The male gametophyte is extremely sensitive to temperature, and its ability to maintain its physiological state under heat stress is known as acquired thermo-tolerance^27^.

The dominant factors that control pH on a European scale are the crystalline substrate (bedrock) in combination with temperature and precipitation^28^. The pH maps, which provide a unique set of homogeneous and spatially representative soil pH data for the European continent^28^, mainly reflect the natural habitat conditions on a European scale. Our finding regarding the critical temperatures of germinating pollen grains (and, by extrapolation, probably to whole plants) may be related to the evolutionary context. The latter is connected with the migration of plants beyond the equator due to climate change and adaptation to the spatial distribution of soil pH as a substitute for high temperatures. In Ref.^7^, the author fitted a beta distribution to some growth rate data, thereby capturing variations concerning temperature and pH, which have a surprising conjugate nature^7^ (see also a comment in the Appendix in Supplementary Information).

Critical temperature measurements can also help provide solutions for the assisted migration of the plant species^29^ that are threatened with extinction in the face of rapid climate change. In this application, the ELoPvC tool (plus the R code provided in Supplementary Information) can be a complementary and competitive (owing to its simplicity and low cost) method for genetic research on assisted migration processes. It would enable the best species from among the species with the appropriate (soil pH, T_c_) dyad to be selected for such an introduction. This method might help breed resilient crops to thermal stress and climate change.

### Using ELoPvC in basic studies

Establishing a precise, non-invasive method for detecting the effect of temperature on the growth of individual plant cells opens up new possibilities in basic research. Last year’s Nobel Prize in Physiology or Medicine was awarded based on the discoveries of temperature and touch receptors. Examining gene expression in response to capsaicin revealed an ion channel protein, the (temperature “equivalent”) capsaicin receptor TRPV1^30^. The ELoPvC method enables the ion fluxes in a single cell to be measured during growth, which allows the influence of temperature on this process to be monitored. The latter makes the ELoPvC technique a promising candidate for research on various mutants in the genes encoding the ion channel proteins to confirm their role in the ion exchange between a cell and the environment during growth. On the other hand, this method can also be used to study the influence of various external factors on cell growth, including treatment with phytohormones or ion channel inhibitors. Combined with single-cell transcriptome sequencing, this technique provides scientists with new tools to study cell growth at the molecular, biochemical, and biophysical levels.

The problem of pollen tube growth has been the subject of intensive research, resulting in the development of state-of-the-art methods and protocols^31^. However, the problem significant for fertilization issues, which are essential for the life of the flowering plants on Earth, of electrical interactions and ion exchange between the elongating cells that leads to competitive or cooperative behavior, has not yet been addressed owing to the lack of an effective method. This report presents a new experimental approach that can be further developed by including higher sampling rates or even better instruments with the nano-voltage range. The problem of pollen tube growth oscillation with simultaneous ion exchange (and many others) can also be investigated with this sensitive, non-invasive technique for a single growing cell, as was already indicated in our earlier work^2^. However, the apparent limitation of this method is the lack of spatial resolution—only the resultant signal is detected.

The results of this work broaden our understanding of the non-equilibrium phase transitions^32^ into living cell systems and clarify the physical dimension of homeostasis, as all regulatory mechanisms are unclear and incomplete unless the system establishes a precise reference temperature (in the Kelvin scale). The phenomenon of collective excitation^33^ in molecular dynamics, well known in condensed matter physics, relates to the synchronous operation of specific ion channels and wave propagation at a critical temperature. It is an essential factor that operates in parallel with the appropriate temperature receptors (coding) to achieve the optimal conditions for intensified metabolic processes, overall cell homeostasis, and extreme growth.

## Conclusions

The article presents a non-equilibrium phase transition at the critical point of a system of freely evolving and interacting pollen tubes. We use the non-invasive contact electrode method to determine the level of the chemical potential for the oscillation of the ions of individual cells with a semiconductor measuring device. The results of the experiments were analyzed using (non-equilibrium) dynamic entropy calculations and several other advanced statistical physics methods to locate the critical temperature of an isolated droplet containing a collection of living pollen tubes. We confirmed the existence of an ionic phase transition in the cell system of growing pollen tubes. We described the role of the short-term instability of the homogeneous (single-site) stochastic dynamics in generating the transition. We used quantitative experimental methods interpreted using the predictive physical theoretical methods. This mixed approach is scientifically compelling for the community interested in studying the emergent properties or non-equilibrium processes in metabolically active biological systems and in the field of (soft) condensed matter physics. Considering the problem addressed in this report is based on a specific physical implementation, we observed the signatures of an emergent macroscopic coherent state (ordered, collective behavior) in a non-equilibrium system of growing cells at critical temperatures. The non-equilibrium phase transition associated with the change in ionic noise generated by living cells in the vital area turns out to be a temperature detector of cell homeostasis not only of plants^3^ but also humans^5^, thus encompassing the kingdom of highly organized life.

## Materials and methods

The measurements were performed in extenso on the elongating pollen tubes of *Hyacinthus orientalis* L. and *Nicotiana tabacum* L. using ELoPvC^2^, which made it possible to observe the extracellular ion fluxes in one time series (for each temperature), which was a sequence of N = 5000 observations. They were recorded at successive time intervals (0.24 s) for 20 min; see Fig. S1 for a visual explanation. The main advantage of this new experimental method is that the system remains intact and grows freely during the entire measurement. Consequently, the system response is not disturbed by the measuring device, which provides a transparent measurement platform.

### Samples

As in Ref.^3^, hyacinths (*Hyacinthus orientalis* L.) were grown under controlled conditions at approx. 25 °C, 30–35% humidity, and high insolation. Fresh pollen grains that were collected from five flowers were immersed in a 2 ml Eppendorf tube containing 0.5 ml of a liquid germination medium (10% sucrose, ten mg/l H_3_BO_3_, 300 mg/l Ca (NO_3_)_2_, 100 mg/l KNO_3_, and 200 mg/L MgSO_4_; pH 6.22 at 23.4 °C). The pollen grains were pre-incubated in an ELPIN + type 357 water bath shaker at 130 rpm for 1.5 h at room temperature in the dark. The research was conducted in the range of physiological growth temperatures for this species (to *calculate* critical growth temperature). The data on tobacco (*Nicotiana tabacum* L., clade: Asterids) were obtained from pollen collected from flowering (Oriental Samsun tobacco) at the Institute of Cultivation, Fertilization and Soil Science, National Research Institute, Puławy, Poland. The rest of the sample preparation and measurement procedure was the same as for the hyacinth but in a different temperature range (see Figs. 1, 2 and 3).

### Electromotive force measurement

The measurement system consisted of an external polystyrene thermostat and an internal Al-coated polystyrene measurement chamber that contained a semiconductor-solute interface (ELoPvC detector^2,5^). The outer casing of the thermostat and the inner shielding box of the measuring chamber was wrapped in a grounded Al shield and black cardboard to provide additional electric and light shielding.

The conductivity measurements of the germination medium (control), which were taken using a CC-105 conductivity meter (Elmetron CC-105, Zabrze, Poland), revealed that the conductance equaled 0.348(9) S/cm in a temperature range of 6.1–31.4 °C, which was required to ensure the proper (electrolytic) conditions during the measurements.

A sample containing the tobacco (*Nicotiana tabacum* L.) or hyacinth (*Hyacinthus orientalis* L.) pollen tubes was placed in a liquid (conductive) germination medium. The selected group of pollen tubes, contained in 20–40 µl germination medium, was transferred onto a photovoltaic semiconductor (n-p, phosphorus–boron, junction on silicon crystal) plate^2,3^ located in a grounded Faraday cage. After the transfer, the system was stabilized for approximately one hour. The DC voltage (digital filter on) was measured in the physiological temperature range, which captured a mean field of a collective of cells at a 4.1 Hz sampling, thus fulfilling the Nyquist sampling criterion for the long-period ion dynamics of a growing pollen tube. We used a DMM 4040 6-1/2 Digit Precision Multimeter from Tektronix, Inc. (Beaverton, OR, USA) and then recorded it as a 20 min time series (N = 5000) on an external disc. The temperature control system consisted of an integrated control circuit^5^ and a 1 W heater (ceramic resistor) or ice, which was added below ambient temperature. The measurements were taken in the dark at approximately 30–40% humidity (a moist cotton pad placed in the inner chamber was used to avoid sample evaporation) in a geomagnetic field of 50 μT. The measurements were feasible owing to the subtle effects—bending of the energy bands—that occurred at the semiconductor-liquid interface^34^. The series of time data collected at each temperature (Fig. S1) using this non-invasive solute-semiconductor interface technique were first detrended (Figs. 5 and 6b). Then they were analyzed using a program written in R (Fig. S2). For control data, see Ref.^3^.

### Analytical methods

Information theory, a field bordering on probability theory, statistics, computer science, and statistical mechanics, provides a spectrum of nonlinear methods to capture the internal structure of a signal with insight into its complex nature. Many ready-made advanced procedures can be found in the R programming language environment. The experimental series of time data, collected at each temperature, were detrended and analyzed using a program written in R^35^ by the author. The nonlinear statistical metrics, namely the Hurst exponent^36^, the largest Lyapunov exponent^37,38^, and the entropy^39,40^ of a measured time series for the detected ion-induced EMFs, were evaluated (Table 1). The (corrected R/S) Hurst exponent is used to measure the long-term memory of a time series. It refers to the autocorrelation of time series and the rate at which it decreases with an increasing delay between the pairs of values^41^. The Lyapunov exponents are the mean exponential coefficients of divergence or convergence of nearby orbits in phase space^37^. The maximum Lyapunov exponent (Λ) describes the speed of trajectory convergence or divergence in each attractor dimension and estimates the amount of chaos in the system^42^. Quantitatively, two trajectories in a phase (or state) space with an initial separation vector $\delta {\varvec{Z}}_{0}$ diverge at a rate given by $\left| {\delta {\varvec{Z}}\left( t \right)} \right| \approx e^{\lambda t} \delta {\varvec{Z}}_{0}$, where λ is the Lyapunov exponent. The dynamic entropy (approximate^39^—ApEn, sample entropy^40^—SampEn) quantifies the size of the fluctuation regularity in a time series (Table 1). A low entropy value indicates that a time series is deterministic, whereas a high value indicates its randomness. Fuzzy entropy^43^ and many others can also be used as a nonlinear measure of time series complexity; e.g., Velichko and Heidari^44^ have recently proposed a method for calculating entropy utilizing an algorithm for artificial neural networks. Entropy, in general, has proven to be a practical function in extracting meaningful information from raw brain waves (see Ref.^45^ for an overview and definitions of entropy). However, a significant difference is that in our experiment, the dynamic variables calculated from the collected data, including entropy, were parameterized by the ambient temperature (thermostat).

The information entropy^18^

$$H\left( X \right) = - \mathop \sum \limits_{i = 1}^{N} p\left( {x_{i} } \right){\text{log}}_{b} p\left( {x_{i} } \right)$$

was calculated from the histogram data (see inset in Fig. 1) in R code at physiological temperatures using the entropy calculation procedure^46^ and then interpolated with the sinc function. The phase space was reconstructed using the Takens theorem^47^. The power spectral density (PSD), calculated by the Welch method^48^ and covered with the results of the discrete Fourier transform (light gray), showed the behavior of $1/f^{\beta } \user2{ }$(flicker) noise for approximately 3 decades of frequency. The spectral signature $\beta$, which corresponded to the fractal dimension of the system dynamics, was determined from the linear slope.

To summarize, the data (in the form of 20-min time series, each series measured at a different temperature, Fig. S1) from the measurements were used for the analysis using the statistical programming language R. The research concerned the net fluctuations that were generated in a group of pollen tubes of the species *Nicotiana tabacum* L. or *Hyacinthus orientalis* L. in the temperature ranges that are characteristic for optimal growth. This article is based on the results that were obtained from the number crunching *calculations* (total: two matrices with dimensions of 11 × 62 of the dynamic variables (682 resulting real numbers) depending on the temperature and 1,116 auxiliary plots: 2 × nine × 62 panels). To the best of our knowledge, the materials that were collected for this publication have no equivalent in the world scientific literature as yet.

### Ethics statement

Author complies with the IUCN Policy Statement on Research Involving Species at Risk of Extinction and the Convention on the Trade in Endangered Species of Wild Fauna and Flora.

## Supplementary Information

Supplementary Information.

## Data Availability

The data supporting this study’s findings are available within this article and its Supplementary Information. Further data are available from the corresponding author upon reasonable request.
