# Supplementary material for: Collective excitations of germinating pollen grains at critical points
Source: Sci Rep. 2023 Jan 12;13:610. doi: 10.1038/s41598-023-27754-6 (PMC9837070; doi:10.1038/s41598-023-27754-6)
Supplement: Supplementary file 1 — Supplementary Information. [file 41598_2023_27754_MOESM1_ESM.docx]

Supplementary Information

**Collective excitations of germinating pollen grains at critical points**

Mariusz A. Pietruszka

Corresponding author. Email: [mariusz.pietruszka@us.edu.pl](mailto:mariusz.pietruszka@us.edu.pl)

**This file includes:**

**Figure S1** Data management

**Figure S2** Software - R code

**Appendix**


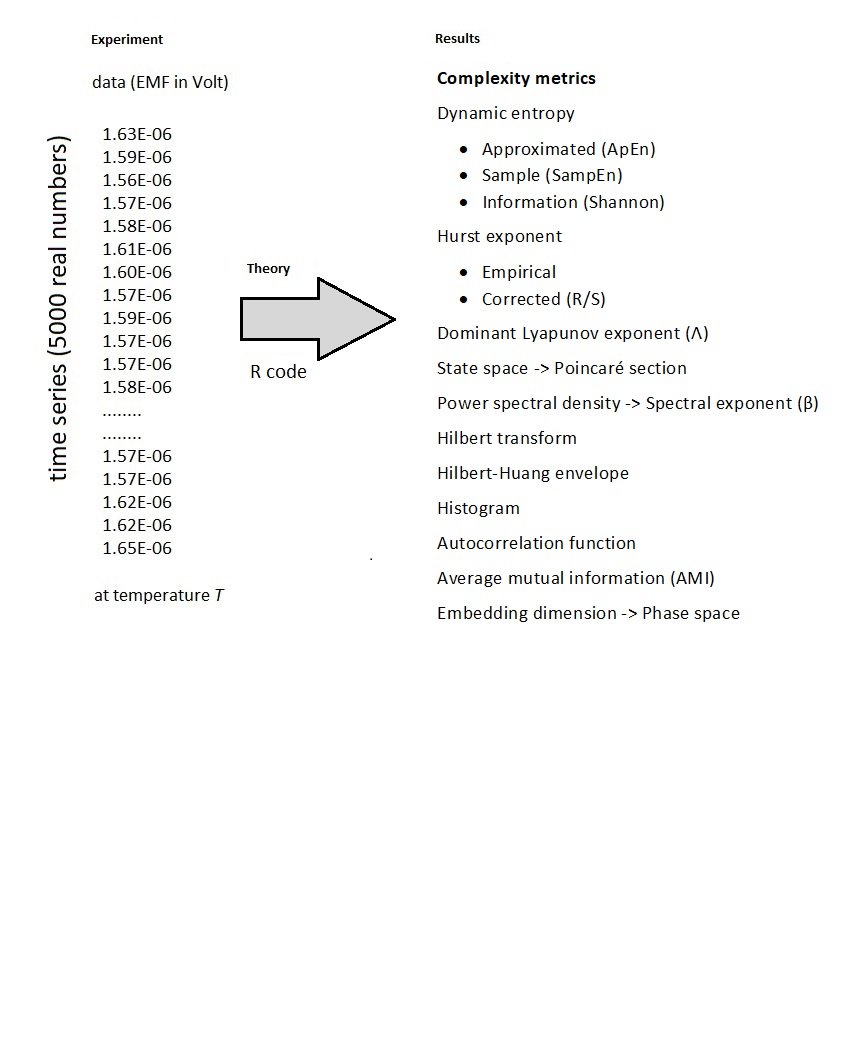


**Figure S1** Data acquisition and data flow diagram for a *single* measurement at temperature *T* (left) exemplary raw data set of 5,000 time points at 4.1 Hz sampling) and (right) the resulting dynamic metrics. Each 20-minute measurement, using time-series regularity metrics for extracellular ion flux data from the pollen tube population, gave the results in the form of real numbers (8) and full graphs (8) after numerical calculations in the R code. The measurement for the fresh pollen tubes was repeated for a number of temperatures within the physiological temperature range in order to obtain graphs such as those presented in Figs. 1-3 (main text). The entire experiment consisted of a series of measurements at different but constant temperatures.

# Software

# Dynamic Metrics program (by MAP, 2021)

# Install packages

# Insert path to the raw data file (here: meas0001.txt); use .txt (ANSI) file

inp<-scan("C:\\Users\\Insert_User\\Desktop\\R-work\\meas0001.txt") # Windows version

# inp<-scan("/home/user/R-work/meas0001.txt") # Linux version

# and Run a program

detrend_inp<-pracma::detrend(inp)

t<-1:5000

x<-detrend_inp

#

par(mfrow = c(1,1))

library(entropy)

hist(x, xlab = "EMF (V)", main = "Histogram of EMF", col='light blue')

h<-hist(x, xlab = "EMF (V)", main = "Histogram of EMF", col='light blue', ylim = c(0, 1600))

text(h$mids, h$counts, labels = h$counts,adj=c(0.5, -0.5))

y = c(1 ,45, 227 ,701, 1529, 1583, 534, 342, 25, 12, 1) # Insert histogram values

#

# Gaussian distribution; can be omitted

hist(x, probability = T, col = 'light blue', main = 'Gaussian Distribution', xlab = "EMF (V)")

lines(density(x), col = 'red', lty = 1, lwd = 2)

#

# Shannon entropy

entropy::entropy.ChaoShen(y)

entropy::entropy.ChaoShen(y, unit = c("log2")) # in bits

entropy::entropy.empirical(y)

#

# ApproxEn; SampleEn

pracma::hurstexp(detrend_inp)

pracma::approx_entropy(detrend_inp)

pracma::sample_entropy(detrend_inp)

#

# Combine plots into a plot array

par(mfrow = c(3, 3))

time<-seq(5000)*0.24

plot(t, inp, main = "EMF - raw data", xlab = "N", ylab = "EMF (V)", cex = .2, pch = 10)

grid(NULL, NULL , lty = 6, col = "cornsilk2")

plot(time, x, main = "EMF - detrended data", xlab = "Time (s)", ylab = "EMF (V)", cex=.2, pch=10)

grid(NULL, NULL, lty = 6,col = "cornsilk2")

#

# time<-seq(5000)*0.24 # Sampling rate [s]

H_T <- hht::HilbertTransform(x)

plot(time, H_T, xlim = c(0., 1200), main="Hilbert Transform", xlab = "Time (s)", ylab = "Voltage (V)", cex = .4, pch = 1)

grid(NULL, NULL, lty = 6,col = "cornsilk2")

lines(time, Re(H_T),col = "green")

lines(time, Im(H_T), col = "red")

# legend(100, 1e-05, col = c("black", "green", "red"), lty = c(NA, 1, 1), pch = c(1, NA, NA), legend=c("Signal", "Real", "Imaginary"), cex = .8)

legend("topleft", inset = 0.02, col = c("green","red"), lty = c(1, 1), pch = c(NA, NA), legend=c("Real", "Imaginary"), cex=0.8, box.lty = 0)

env<-hht::HilbertEnvelope(x)

plot(time, env, col = "red", xlim = c(0, 1200), main = "Hilbert-Huang Envelope", xlab = "Time (s)", ylab = "Voltage (V)", cex =.1, pch =10)

grid(NULL, NULL, lty = 6,col = "cornsilk2")

hist(x, xlab = "EMF (V)", main = "Histogram of EMF", col = 'light blue')

suppressMessages(library('nonlinearTseries'))

library(plot3D)

d_i.x=detrend_inp

tau.acf = timeLag(d_i.x, technique="acf", lag.max = 100, do.plot = T, xlab="Time lag (s)", main = "Autocorrelation Function")

# acf<-acf(d_i.x, type = "covariance")

tau.ami = timeLag(d_i.x,technique = "ami", lag.max = 100,do.plot = T, xlab = "Time lag (s)")

emb.dim = estimateEmbeddingDim(d_i.x, time.lag = tau.ami, max.embedding.dim = 15, xlab = "Dimension (d)")

tak = buildTakens(d_i.x, embedding.dim = emb.dim, time.lag = tau.ami)

scatter3D(tak[,1], tak[,2], tak[,3], main="Reconstructed phase space", col = 1, type = "o", cex=0.2)

# End

**Figure S2** A ready-to-use (standalone) computer program (R code).

**Appendix**

For facilitating plant migrations due to climate change, see also:

Kosek, J., and Pietruszka, M. (2022). An equation for determining the beta Euler shape exponents of plant growth data – a program toolbox for plant physiologists. Preprint available at Research Square: <https://doi.org/10.21203/rs.3.rs-1920863/v1>

containing a Python/SAGE program.
